# Combining metformin with lactate transport inhibitors as a treatment modality for cancer - recommendation proposal

**DOI:** 10.3389/fonc.2022.1034397

**Published:** 2022-10-24

**Authors:** Don Benjamin, Michael N. Hall

**Affiliations:** Biozentrum, University of Basel, Basel, Switzerland

**Keywords:** cancer, metformin, monocarboxylate transporter, lactic acid, MCT4, syrosingopine

## Abstract

Highly glycolytic cancer cells excrete lactate to maintain cellular homeostasis. Inhibiting lactate export by pharmacological targeting of plasma membrane lactate transporters is being pursued as an anti-cancer therapy. Work from many laboratories show that the simultaneous inhibition of lactate export and mitochondrial respiration elicits strong synthetic lethality. The mitochondrial inhibitor, metformin, has been the subject of numerous clinical trials as an anti-cancer agent. We propose that, in future clinical trials, metformin be combined with lactate transport inhibitors to exploit this synergistic interaction.

## Introduction

Metformin is widely prescribed for management of type 2 diabetes (83 million prescriptions in the US for 2018) and is well-tolerated with minor side-effects in non-contraindicated patients. It inhibits complex I of the mitochondrial electron transport chain leading to lowering of blood glucose levels. The wealth of epidemiological data for metformin, due to its extensive use, has shown that diabetic patients on metformin have a lower incidence of cancer (1). An anti-cancer activity of metformin was subsequently demonstrated in a variety of pre-clinical models. This triggered a flood of clinical studies investigating metformin for potential anti-cancer efficacy (over 300 listed in clinicaltrials.gov). The trials evaluate metformin for many uses, including for cancer prevention, neoadjuvant applications in chemo- or radiotherapy, and first-line therapy. Based on completed trials, the anti-cancer activity of metformin is weak with little-to-no clinical benefit (albeit with many trials still in progress). An ongoing controversy is that serum levels of metformin attained with the standard dose used in these trials (guided by diabetes dosing) are significantly below that used to demonstrate anti-cancer activity in pre-clinical models. Proposals for achieving clinical anti-cancer efficacy have included increasing the metformin dose and using the more potent metformin derivative phenformin (2). Nonetheless, metformin does provide anti-cancer clinical benefit in some cases, and it is therefore desirable to lower the effective concentration of metformin required to achieve greater efficacy. We propose combining metformin and a lactate transport inhibitor to achieve greater anti-cancer efficacy.

## Lactic acid production and export in cancer cells

Cells generate energy by metabolizing glucose *via* glycolysis to produce ATP. Under normal conditions, cells divert pyruvate, the end product of glycolysis, into the mitochondria to generate more ATP *via* oxidative phosphorylation. However, in situations where the oxygen supply is restricted, such as during intensive exercise, pyruvate is instead reduced to lactate by LDH (lactate dehydrogenase).

A near-universal metabolic adaptation in cancer cells is a shift from mitochondrial respiration to aerobic glycolysis, the famous Warburg effect, for ATP generation. This re-wiring of basic cell metabolism results in production of excessive amounts of lactic acid, the end product of aerobic glycolysis. Cancer cells thus need to efficiently excrete lactate and protons to avert end-product inhibition of LDH, and to prevent intra-cellular acidification. Lactate/H^+^ export is performed by the monocarboxylate transporters (SLC16 family of solute carriers) across the plasma membrane. The most relevant isoforms in cancer biology are MCT1 (SLC16A1), which is ubiquitously expressed and has high affinity for lactate and pyruvate, and MCT4 (SLC16A3) (3). MCT4 is normally restricted to glycolytic tissues such as white skeletal muscle, astrocytes and activated macrophages. However, its expression is induced by HIF-1α under hypoxic conditions common to tumors. MCT4 is thus present in most tumors, regardless of tissue of origin, and indicates poor prognosis (4). MCT4 has a lower affinity for lactate compared to MCT1, effectively restricting it to lactate export, but has a high transport capacity (5). This role as a high-capacity transporter dedicated to lactate export makes MCT4 a promising cancer drug target.

## MCT inhibition in cancer therapeutics

As lactate export assumes a greater importance in cancer cells, this has spurred the development of MCT1 and MCT4 inhibitors as anti-cancer drugs. An MCT1-specific inhibitor, AZD3965, is in phase 1 trials as a first-in-human and first-in-class agent in patients with solid tumors (https://www.clinicaltrials.gov/ct2/show/NCT01791595). AZD3965 was well-tolerated with demonstrated target engagement at administered doses, and a recommended phase 2 dose (RP2D) has been proposed.

AZD3965 has no inhibitory effect on MCT4, limiting its usefulness to tumors where MCT1 is the sole effective lactate transporter. Indeed, an inclusion criterion of the above-mentioned AZD3965 trial was for patients with tumors expressing high MCT1 and low-to-no MCT4. This is a severe limitation as MCT4 is present in most tumors (3, 6). Despite considerable effort, no MCT4-specific inhibitor has entered clinical testing. Nonetheless, a handful of established drugs have been identified as having an inhibitory effect on MCT4. The anti-hypertensive drug syrosingopine was recently identified as a dual MCT1/MCT4 inhibitor (7). At higher concentrations, the analgesic diclofenac and lipophilic statins have also been reported to inhibit MCT4 (8–10). These drugs are well-characterized with respect to their clinically approved targets, but were later shown to have an off-target effect on lactate transport. Thus, there are several approved pharmacological agents that can be re-positioned for clinical trials to test their suitability and efficacy for off-label use in cancer.

## Combining MCT inhibitors with metformin

Inhibiting lactate export, by itself, is not particularly deleterious to cancer cells. However, blocking lactate export can be leveraged to elicit cell killing (11, 12). Inhibition of mitochondrial oxidative phosphorylation with metformin reduces ATP production. Due to metabolic flexibility, cells can shift from oxidative phosphorylation to aerobic glycolysis for compensatory ATP production (the reverse can also occur when glycolysis is inhibited) (13). The increase in glycolysis upon metformin administration results in increased lactate production. This creates a dependence on lactate export that can be exploited as a vulnerability.

Accumulation of intracellular lactate, upon inhibition of lactate export, impedes LDH activity. The reactions catalyzed by LDH and the mitochondrial complex 1 (the target of metformin) are both coupled to the oxidation of NADH to NAD+. Indeed, these reactions are the main sources of NAD+ regeneration. NAD+ is required for ATP production by glycolysis. NAD+ and NADH are also essential co-factors in many redox reactions and a proper NAD+/NADH ratio is important for cellular homeostasis. Thus, simultaneously blocking lactate export and complex 1 activity causes an acute drop in NAD+ levels resulting in synthetic lethality. The importance of synthetic lethality is underscored by the observation that, due to autoregulation of lactate efflux, merely blocking lactate transporters alone is not sufficient to reduce glycolytic flux to lactate (14).

This synthetic lethality has been independently reported in multiple studies. MCT1 and/or MCT4 function was inhibited genetically (15) or pharmacologically (7, 16, 17), and lactate export-inhibited cells were further challenged with mitochondrial inhibitors. This resulted in cell death that was not observed with either inhibitor alone. The mitochondrial respiration inhibitors employed in these studies were metformin and phenformin, an obvious choice due to their suitability for potential clinical application. A newly described MCT4 inhibitor, VB124, also elicited synthetic lethality with phenformin (18). There is therefore ample demonstration that inhibition of lactate transport elicits synthetic lethality in combination with inhibition of mitochondrial respiration. Our own observations, as summarized in **Table 1**, show a substantial shift in the *in vitro* IC50s for syrosingopine and AZD3965 when combined with metformin. The efficacy of AZD3965 and syrosingopine is consistent with MCT1 and MCT4 expression status. AZD3965 treatment suppresses growth in an MCT1-dependent cell line (K562), but is ineffective when MCT4 is expressed (HL60 and SkBr3). Syrosingopine, however, effectively suppresses growth in all 3 cell lines by virtue of its dual MCT1/MCT4 inhibitory properties. In all cases, however, adding metformin on top of the lactate transport inhibitor (AZD3965 or syrosingopine) greatly reduces cell numbers by eliciting cell death.

**Table 1:** *In vitro* IC50 of syrosingopine (S) and AZD3965 (AZ) in a panel of human cancer cell lines with varying MCT1 and MCT4 expression status.

	S	S+M	AZ	AZ+M
	IC50	IC50	IC50	IC50
**HL60** **(MCT1+, MCT4+)**	10μM	2μM	>1 μM	>1 μM
**K562** **(MCT1+, MCT4-null)**	10μM	2μM	0.1μM	0.01 μM
**SkBr3** **(MCT1-null, MCT4+)**	10μM	0.1μM	>1 μM	>1 μM

IC50 shows the half-lethal concentration of each drug in growth assays (4 days) either alone or in combination with metformin (M). Metformin was used at a concentration of 4mM where it has a negligible effect on cell growth. Data is compiled from multiple experiments.

The outcome of the above studies is that while lactate transport inhibitors and metformin separately have only a mild effect on the viability of cancer cells, together they combine to induce cell killing. Indeed, in view of the interest in clinical use of metformin for cancer, it is notable that syrosingopine enhances the anti-cancer activity of metformin approximately 15-fold, as measured by a corresponding decrease in the metformin IC50 (11).

## Discussion

We propose combining metformin with lactate transport inhibitors as a treatment modality in cancer. The widespread use of metformin as an anti-diabetic has resulted in a wealth of clinical data on its safety. This has in turn led to willingness in the medical community for its prescription, even among non-diabetics, for anti-cancer therapy.

Syrosingopine was previously in clinical use for over 4 decades as an anti-hypertensive drug, until it was retired in favor of better anti-hypertensives. It was expressly developed as a mild version of its parent drug reserpine, and was administered to patients daily for years (similar to metformin for diabetes). It has a low incidence of adverse events, with the most commonly reported adverse effect being lethargy, headache, gastrointestinal upset, vertigo and nasal congestion. The clinical history of syrosingopine indicates an acceptable safety profile for off-label prescription as a clinic-ready dual MCT1/MCT4 inhibitor. Due to syrosingopine’s primary role as an anti-hypertensive, a reduction in blood pressure should be monitored. Potential consequences of MCT1/4 inhibition may arise in tissues where lactate export plays a role in normal physiology. This could be the case in skeletal muscle after exercise, or in the brain where glial cells secrete lactate to feed neurons (syrosingopine crosses the blood-brain barrier). Indeed, lethargy and headache are among the reported adverse effects of syrosingopine. Brain cells however, have high expression of MCT2 which is not inhibited by syrosingopine (7). However, notwithstanding the safety records of both drugs taken individually, it would be prudent to monitor any unexpected consequences from their combined administration. We note that we did not observe signs of acute toxicity in treated mice. Furthermore, the drug combination had no effect on untransformed cells in culture (11).

Despite high initial expectations raised by epidemiological data and *in vitro* pre-clinical models, metformin has performed underwhelmingly in most cancer related trials. As mentioned, the low serum metformin levels attained in these clinical trials may be insufficient to elicit anti-cancer activity.

It would therefore be desirable in trials of lactate transport inhibitors that metformin be included in the trial protocol, or vice versa, to exploit any synthetic lethality elicited by the combination. Metformin has shown modest clinical benefit in some trials, and syrosingopine is experimentally proven to significantly reduce the concentration of metformin required for cancer cell killing. Thus, inhibiting lactate export is a rational strategy to potentiate anti-cancer activity of metformin in future clinical trials.

## Data availability statement

The original contributions presented in the study are included in the article/supplementary material. Further inquiries can be directed to the corresponding author.

## Author contributions

DB wrote the manuscript. MH edited and approved the final manuscript. All authors contributed to the article and approved the submitted version.

## Conflict of interest

The authors declare that the research was conducted in the absence of any commercial or financial relationships that could be construed as a potential conflict of interest.

## Publisher’s note

All claims expressed in this article are solely those of the authors and do not necessarily represent those of their affiliated organizations, or those of the publisher, the editors and the reviewers. Any product that may be evaluated in this article, or claim that may be made by its manufacturer, is not guaranteed or endorsed by the publisher.
